# A20 Controls Macrophage to Elicit Potent Cytotoxic CD4^+^ T Cell Response

**DOI:** 10.1371/journal.pone.0048930

**Published:** 2012-11-07

**Authors:** Lifeng Wang, Bangxing Hong, Xiaoxia Jiang, Lindsey Jones, Si-Yi Chen, Xue F. Huang

**Affiliations:** Norris Comprehensive Cancer Center, Department of Molecular Microbiology & Immunology, University of Southern California Keck School of Medicine, Los Angeles, California, United States of America; University of California San Francisco, United States of America

## Abstract

Emerging evidence indicates that CD4^+^ T cells possess cytotoxic potential for tumor eradication and perforin/granzyme-mediated cytotoxicity functions as one of the important mechanisms for CD4^+^ T cell-triggered cell killing. However, the critical issue is how the cytotoxic CD4^+^ T cells are developed. During the course of our work that aims at promoting immunostimulation of APCs by inhibition of negative regulators, we found that A20-silenced Mф drastically induced granzyme B expression in CD4^+^ T cells. As a consequence, the granzyme-highly expressing CD4^+^ T cells exhibited a strong cytotoxic activity that restricted tumor development. We found that A20-silenced Mф activated cytotoxic CD4^+^ T cells by MHC class-II restricted mechanism and the activation was largely dependent on enhanced production of IFN-γ.

## Introduction

CD8^+^ T cells are the most cytotoxic T lymphocytes (CTLs) that directly destroy virus-infected or malignant cells. CD4^+^ T cells are recognized for their coordinated orchestration by production of various cytokines, such as T helper (Th)1 producing interferon (IFN)-γ to promote cellular immunity, Th2 producing interferon (IL)-4 to potentiate humoral immune response, and Th17 producing IL-17 to facilitate inflammation and autoimmune diseases. Recent studies further identified different subsets of CD4^+^ regulatory T cells which perform immune regulation on effector T cells by expressing transcription factor FoxP3 or by secreting anti-inflammatory cytokine IL-10 or transforming growth factor (TGF)-β. However, emerging evidence indicates that CD4^+^ T cells also develop cytotoxic activity to directly participate in cytolysis of tumor or infected cells. For instance, tumor-reactive CD4^+^ T cells were found to develop cytotoxic activity and eradicate large established melanoma after transfer into lymphopenic hosts [1], [2]. The critical issue is how these cytotoxic CD4^+^ T cells are developed.

Macrophages (Mфs) are initially recognized as phagocytic cells responsible for pathogen elimination and housekeeping function in homeostasis and tissue repair. The classically known Mфs, which are activated by microbial products or interferon (IFN)-γ, produce large amounts of proinflammatory cytokines, express high levels of MHC molecules, and function as a potent killer of pathogens and tumor cells [3]. Dependent on the anatomical location and the physiological or pathological context, Mфs can be alternatively activated by anti-inflammatory cytokines such as IL-4 or IL-13 [4]. The alternatively activated Mфs produce high amounts of IL-10, express scavenger receptors, and exhibit anti-inflammatory and tissue repair functions [5]. Recent studies suggest that Mфs represent a very plastic cell population that play an essential role in the regulation of the pro-inflammation vs anti-inflammation and in the coordination of the pro-tumorgenesis vs. anti-tumorgenesis [6]. Classically activated Mфs and alternatively activated Mфs represent two extremes in the spectrum of the phenotype and functionality of Mфs [5], [7].

To promote the antitumor activity of Mф, we used an A20 silencing strategy to enhance the classical activation of Mф. This was based upon the published studies that A20, a zinc-finger ubiquitin-modifying enzyme, inhibits several upstream signaling pathways of NF-kB in a feedback manner by degradation or deactivation of signaling molecules via its dual functions of ubiquitination and deubiquitination [8], [9], [10]; A20-deficient Mфs display prolonged NF-κB activity [8], [10]; A20-silenced dendritic cells (DCs) express higher levels of costimulatory molecules and proinflammatory cytokines, and display a superior immunostimulatory ability [11]. We found that A20-silenced Mф not only enhances expression of perforin and granzyme B in CD8^+^ T cells and Natural Killer (NK) cells, also drastically upregulate these cytotoxic molecules in CD4^+^ T cells. As a consequence, the granzyme-highly expressing CD4^+^ T cells exhibited cytotoxic activity in vitro/vivo. We further defined that A20-silenced Mф activated cytotoxic CD4^+^ T cell response by MHC class-II restricted mechanism, and the activation was largely dependent on enhanced IFN-γ production.

## Results

### A20 Controls Mф Maturation and Immunostimulatory Activity

**Figure 1:**
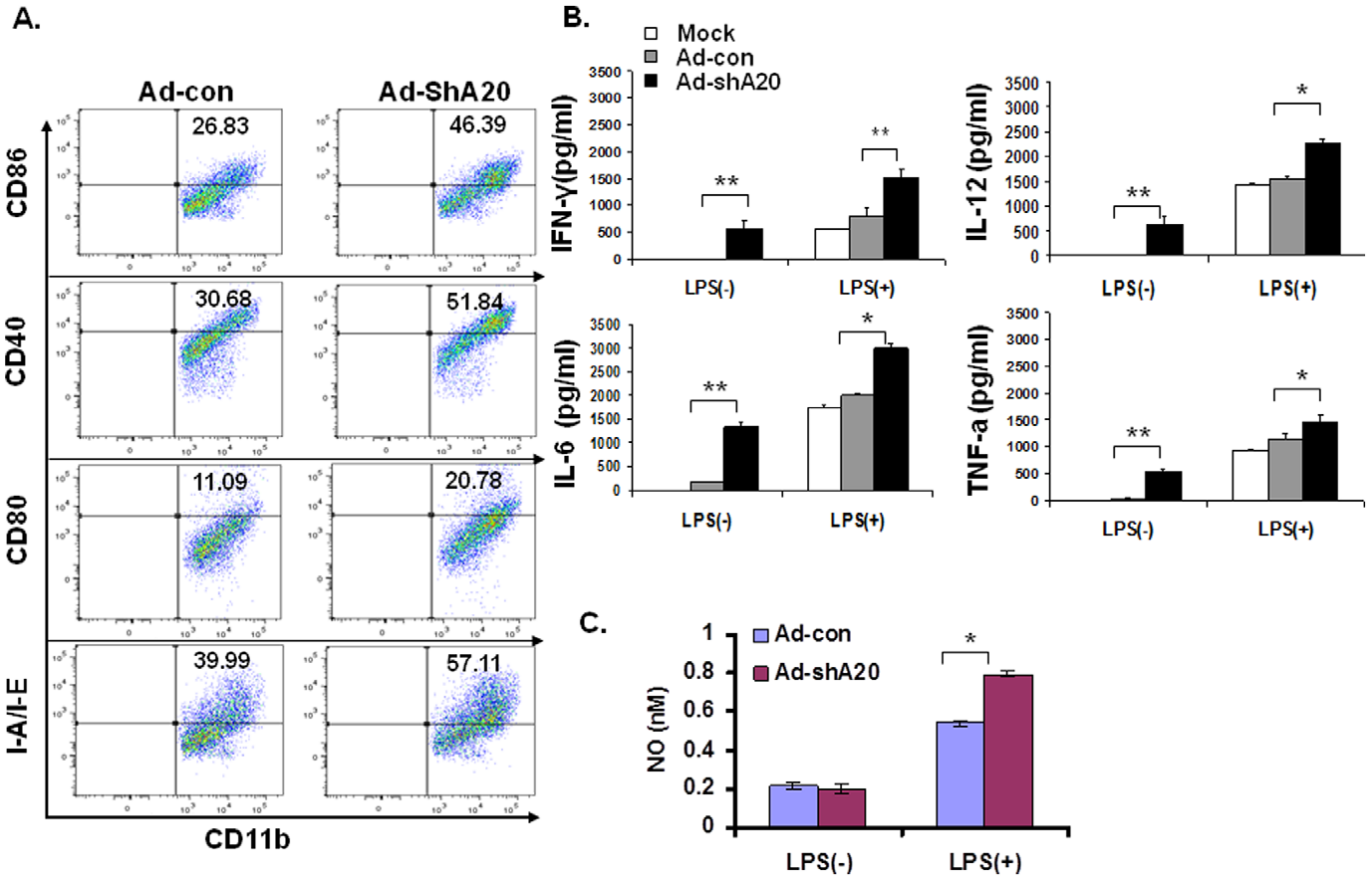
A20 controls maturation and cytokine production of Mф. **A**. Expression of costimulatory molecules and MHC class II molecule on the adenoviral-transduced BMMф in response to stimulation of LPS. **B**. Production of inflammatory cytokines by the adenoviral-transduced BMMфs, as tested by ELISA. **C**. NO production by adenoviral-transduced BMMфs, as tested by Griess assay. Experiments were repeated three times with similar results. *p<0.05, **p<0.01 Ad-shA20- vs. Ad-con-transduced Mф.

To investigate whether A20 controls maturation of Mф, bone morrow-derived Mфs (BMMфs) were transduced with adenovirus Ad-A20shRNA (Ad-shA20) or Ad-GFPshRNA (Ad-con). Downregulation of A20 expression by Ad-shA20 was confirmed via quantitative RT-PCR (qRT-PCR) at the level of mRNA and via intracellular staining (ICS) at the level of protein (**Fig.S1A&B**). Flow cytometric assay shows that Ad-shA20-transduced BMMфs expressed higher levels of CD80, CD86, CD40 and MHC class-II molecule I-A/I-E than Ad-con-BMMфs under the stimulation of LPS (**Fig.1A** **)**. ELISA results show that Ad-shA20-BMMфs, but not Ad-con-BMMфs, spontaneously produced large amounts of inflammatory cytokines such as IL-6, TNF-α, IFN-γ and IL-12p40, and produced larger amounts of these cytokines in response to LPS stimulation (**Fig.1B** **)**. Adenoviral vector which induces maturation of antigen-presenting cells per se [12] may contribute to the observed “spontaneous” cytokine production by A20-silenced BMMфs. A20-silenced BMMфs also produced higher level of nitric oxide than the control Mфs (**Fig.1C**). Despite the reported anti-apoptotic role of A20 in TNF-treated cells [9], A20-silenced BMMфs showed a comparable viability to Ad-con-BMMфs in cell culture (**Fig.S2**). Taken together, these results imply that A20 negatively regulates the maturation and cytokine production of BMMфs.

Next, we tested if A20-silenced BMMфs possess an enhanced immunostimulatory activity. The transduced BMMфs were pulsed with H2-K^b^–restricted OT-I (SIINFEKL) or OT-II (ISQAVHAAHAEINEAGR) peptide and then co-cultured with CD8^+^ OT-I or CD4^+^ OT-II cells isolated from Ovalbumin (OVA)-specific TCR transgenic mice. Results showed that CD8^+^ OT-I cells cocultured with A20-silenced BMMфs expressed enhanced levels of CD25 and CD44 in comparison with those cocultured with the control BMMфs (**Fig.S3, left**). Moreover, the cocultured OT-I cells with A20-silenced BMMфs produced higher levels of IFN-γ and TNF-α (**Fig.S3, right**) In parallel, A20-silenced BMMфs also more potently activated CD4^+^ OT-II cells, as evidenced by enhanced expression of CD25 and CD69, and heightened production of IFN-γ by the OT-II cells cocultured with Ad-shA20-BMMфs (**Fig.S4**). A20-silenced BMMфs also modestly enhanced proliferation of both CD8^+^ OT-I or CD4^+^ OT-II cells, as tested by ^3^H-Thymidine Incorporation Assay (data not statistically significant and not shown). These results support that A20-silencing endowed BMMфs with an enhanced immunostimulatory activity.

### A20 Controls Mф to Elicit a Cytotoxic CD4^+^ T Cell Response

**Figure 2:**
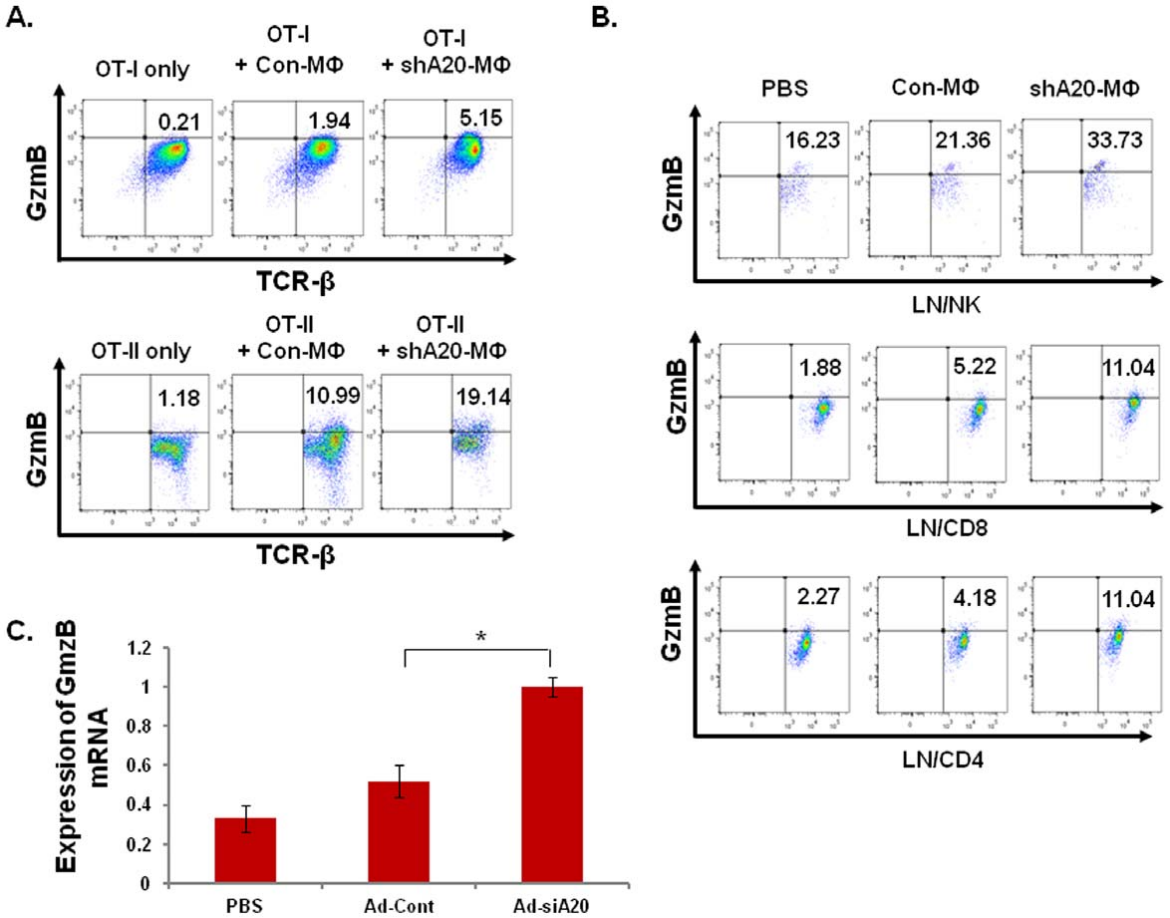
A20-silenced Mф enhances expression of granzyme B in CD4^+^ T cells, CD8^+^T cells or NK cells. **A,** adenoviral-transduced BMMфs were cocultured with freshly isolated OT-I (**uppe**r) or OT-II cells (**lower**) at a raito of 1∶10. 3–5 days later, the cocultured T cells were harvested for analyzing expression of granzyme B by ICS. The data is shown as a representative of 3 independent experiments. (p<0.05, OT-I/shA20-Mф vs. OT-I/con- Mф; p<0.01, OT-II/shA20-Mф vs. OT-II/con-Mф). **B,** C57BL/6 mice (5–6 mice/group) were immunized (*i.p*) twice with different adenoviral-transduced Mфs or PBS. Lymphocytes were isolated from the inguinal LNs to analyze expression of granzyme B in NK cells, CD8**^+^** or CD4**^+^** T cells by ICS. **C.** C57BL/6 mice were immunized (*i.p*) twice with OT-II-pulsed, different adenoviral-transduced BMMфs or PBS. Splenocytes were harvested and in vitro restimulated with OT-II peptide for 48 hrs. CD4^+^ T cells were isolated for analysis of granzyme B expression by qPCR. The data is shown as a representation of three independent experiments. (* p<0.01, shA20- Mф-mice vs. con- Mф-mice).

We examined the potential of A20-silenced BMMф to activate cytotoxic cell responses by testing expression of cytotoxic molecules in the cocultured T cells by ICS. As shown in **Fig.2A**, A20-silenced BMMф enhanced expression of granzyme B in co-cultured CD8^+^ OT-I T cells (**upper**), but also significantly enhanced granzyme B expression in co-cultured CD4^+^ OT-II cells (**lower**). In the meantime, we also detected an enhanced expression of perforin in these co-cultured T cells with A20-silenced BMMф (**Fig. S5**). To rule out that the observed result is derived from the adenoviral transduction of Mф, BMMфs were nucleofected with recombinant plasmid pshuttle-shA20 or pshuttle-shGFP according to the manufacturer’s instruction (Amaxa), which reached ∼40% transfection efficiency, as monitored by Ad-GFP nucleofection in parallel (data not shown). The nucleofected BMMфs were then co-cultured with freshly isolated OT-II T cells in the presence of the OT-II peptide. ICS assay showed that pshuttle-shA20-nucleofected BMMфs display a more potent ability to elicit expression of granzme B in the cocultured OT-II cells (**Fig.S6**). Furthermore, we also tested the potential of A20-silenced BMMф immunization to induce cytotoxic cell responses in mouse model. C57BL/6 mice were i.p. immunized with OT-I/OT-II peptides-pulsed, Ad-shA20 or Ad-con-transduced BMMфs or PBS twice. 7–10 days after the 2^nd^ immunization, spleens and lymph nodes (LNs) were harvested to analyze granzyme B expression in effector cells by ICS. In agreement with the in vitro study, ICS assay explored that A20-silenced BMMфs significantly enhanced expression of granzyme B and perforin in CD4^+^ and CD8^+^ T cells as well as NK cells derived from inguinal lymph nodes (LNs) (**Fig.2B** **& Fig. S5**) or spleen (**data not shown**) of the immunized C57BL/6 mice. qPCR assay further confirmed an enhanced level of granzyme B expressed in CD4^+^ T cells derived from OT-II (not OT-I)-pulsed, A20-silenced BMMф-immunized mice **(** **Fig.2C**). To exclude the possibility that the OT-I/OT-II-pulsed, A20-silenced BMMфs have any different propensity of releasing the loaded antigen to endogenous APCs, we in vitro cultured OVA protein-pulsed, differently transduced BMMфs for one or three days. ELISA analysis revealed that an identical amount of cell-free OVA protein is present in the culture media of differently transduced or Mock BMMфs (data not shown).

**Figure 3:**
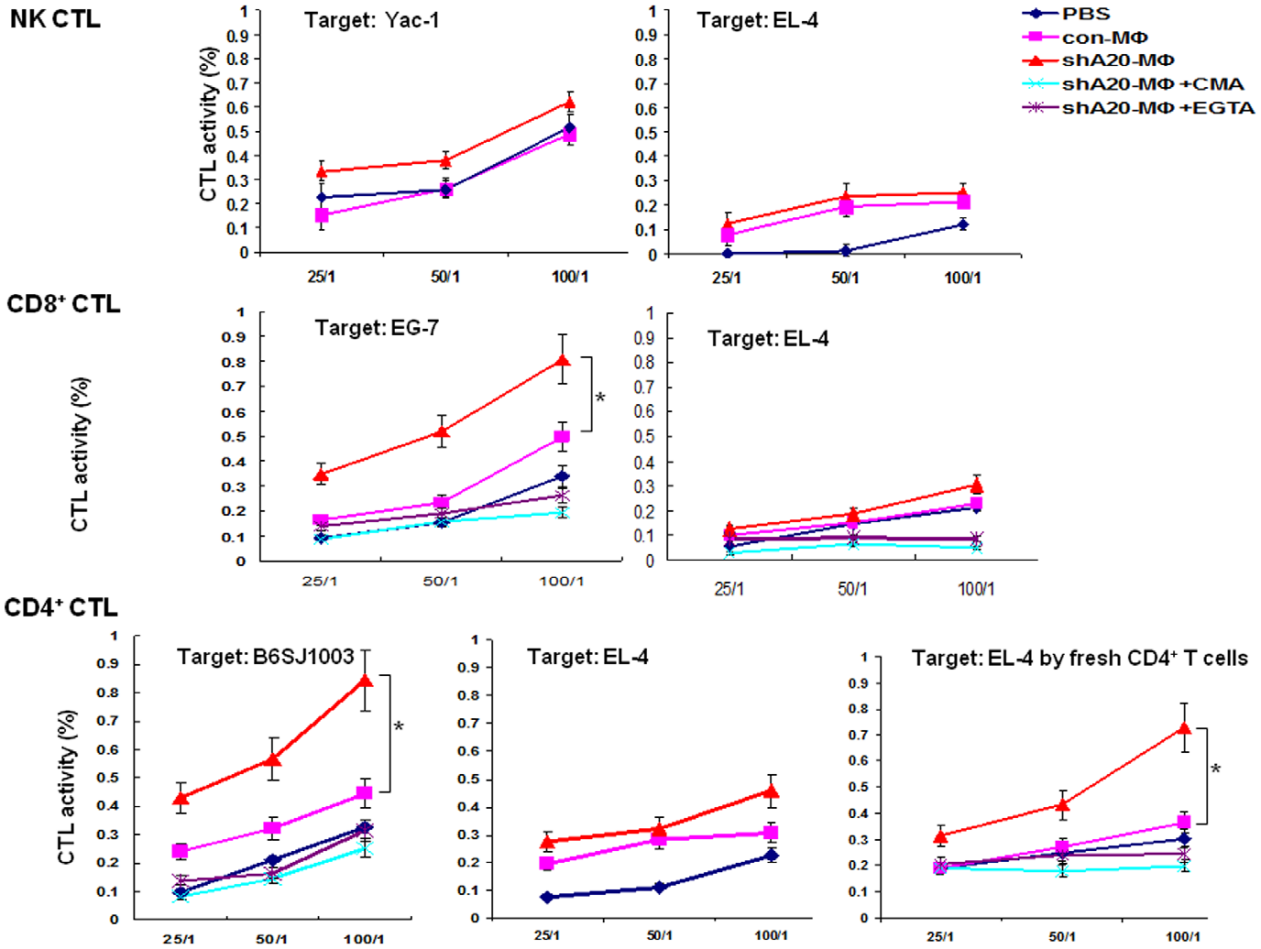
A20-silenced Mф immunization enhances NK cell-, CD8^+^ T cell- and CD4^+^ T cell-mediated cytotoxicity. Splenocytes pooled from 2–3 immunized mice were cultured overnight for NK-mediated cytotoxicity assay or 5–6 days in the presence of OT-I or OT-II peptide for T cells-mediated cytotoxicity assay. The splenocytes cultured with OT-II peptide were selected using anti-CD4 beads prior to cytotoxicity assay. Cytotoxic activities were analyzed by LDH release assay as described in Material and Methods. Experiments were repeated three times with similar results. *p<0.05, Ad-shA20-Mф immunization vs. Ad-con-Mф immunization for specific killing.

To determine cytolytic activity of these effector cells, the splenocytes were isolated from the immunized mice and cultured overnight for the NK-mediated cytotoxicity assay or 5–6 days in the presence of OT-I or OT-II peptide for CD8^+^ or CD4^+^ T cell-mediated cytotoxicity assay. Due to the low expression of MHC class-II molecule on the targeted cell**, a** murine Burkitt lymphoma cell line B6SJ003, the splenocytes cultured with OT-II peptide were selected using anti-CD4 beads prior to the cytotoxicity assay. As shown in **Fig.3**, A20-silenced BMMф immunization enhanced the activity of NK cells, CD8^+^ T cells, and CD4^+^ T cells in killing their specific target cell compared with control BMMф or PBS immunization. The killing specificity of CD8^+^ T cells and NK cells was confirmed by failure of the cytotoxic cells to kill the irrelative control, such as EL-4 cells. We also found that freshly isolated CD4^+^ T cells from A20-silenced BMMф-immunized mice displayed a relatively high non-specific cytolytic activity against the target cell EL-4, but the in vitro culture of these CD4^+^ T cells in the presence of OT-II peptide 5–6 days led these cells to largely lose their non-specific killing activity. Concanamycin A (CMA) acidifies intracellular vacuolar granules to degrade the content in the exocytotic granules [13]. Ethyleneglycotetracetic acid (EGTA) chelates extracellular free calcium to inhibit exocytosis of cytolytic granules and pore formation by perforin [14]. To confirm the CD4^+^ T cell-associated cytotoxicity is mediated by cytotoxic molecules, CMA and EGTA were included for blocking perforin/granzyme activity in some of those cocultures. Data showed that both CMA and EGTA drastically reduced the cytotoxic activity of CD4^+^ T cells (both specific and non-specific), as well as that of CD8^+^ T cells derived from A20-silenced BMMф-immunized mice. Moreover, we also directly demonstrated the role of granzyme B in CD4^+^ T cell-mediated cytotoxicity in the A20-silenced BMMф-immunized mice. OT-II (not OT-I)-pulsed, differently transduced BMMфs were used to immunize C57BL/6 mice and splenocytes were harvested for CTL assay after the 2^nd^ immunization. Result showed that CD4^+^ T cells derived from the A20-silenced BMMф-immunized mice killed OVA-expressing B6SJ003 with a higher efficiency, however, Z-AAD-CMK, a weak and specific granzyme B inhibitor, reduced the CD4^+^ T cells-mediated CTL activity when included into the coculture of OVA-B6SJ003 and CD4^+^ T cells derived A20-silenced BMMф-immunized mice in the CTL assay (**Fig. S7**). The results strengthen our contention that the expressed cytotoxic molecules contribute to CD4^+^ T cell-mediated cytotoxicity, as they do in CD8^+^ T cell-mediated killing.

### A20 Controls Mф to Trigger CD4^+^ T Cell-mediated Anti-tumor Immune Protection

**Figure 4:**
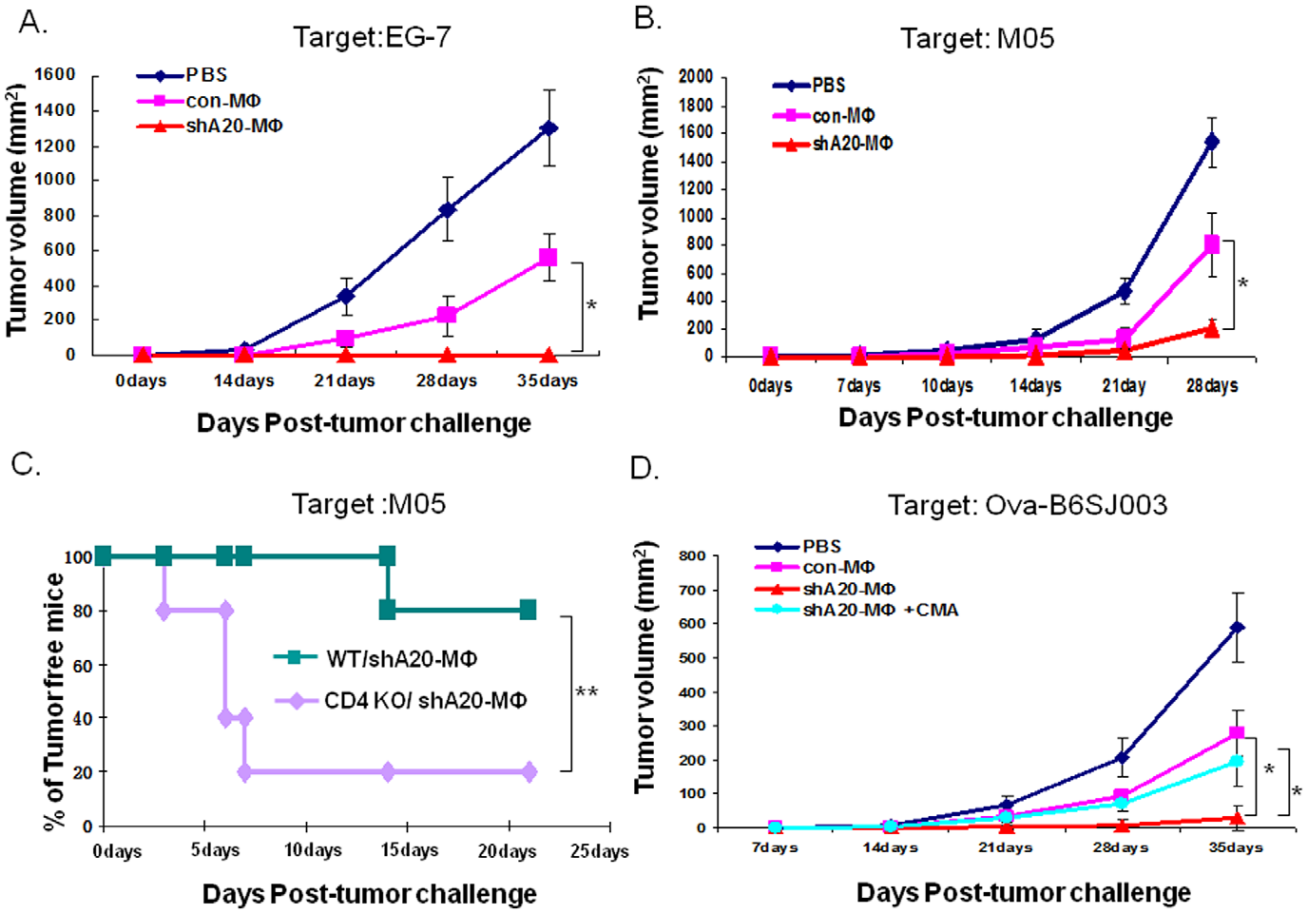
A20-silenced Mф immunization induces enhanced immune protection. **A & B.** C57BL/6 mice (5–6 mice/group) were immunized twice. The mice were s.c. injected with 5×10^5^ EG-7 (**A**) or M05 (**B**). Tumor growth was monitored on the indicated days. * p<0.05, Ad-shA20-Mф immunization vs. Ad-con-Mф immunization. **C**. CD4^−/−^ C57BL/6 or the wildtype littermates (5–6 mice/group) were immunized with OT-II-peptide-pulsed, Ad-shA20-transduced BMMфs twice followed by s.c. injection of 5×10^5^ M05 tumor cells. Tumor occurrence and growth were monitored on the indicated days. **p<0.01, wild-type mice vs. CD4^−/−^ mice. **D**. Transferred OT-II-specific immune pretection. In vitro primed OT-II T cells (5×10^6^) were transplanted into naïve RAG^−/−^C57BL/6 mice (5 mice/group) by retro-orbital injection following s.c injection of OVA-expressed B6SJ1003 tumor cells (6×10^5^). The transplantation of OT-II T cells was repeated one week later. One group of mice were transplanted with CMA-treated, Ad-shA20-transduced Mф-primed OT-II T cells. Tumor growth was monitored on the indicated days. *p<0.05, Ad-shA20-Mф-primed OT-II T cell transfer vs. Ad-con-Mф-primed OT-II T cell transfer, or Ad-shA20-Mф-primed OT-II T cell transfer vs. Ad-shA20-Mф-primed OT-II T cell+ CMT transfer. All the experiments were repeated with similar results.

C57BL/6 mice were immunized with OT-I/OT-II-pulsed, control BMMф or A20-silenced BMMф, or PBS. The immunized mice were challenged with EG-7 tumor cells two weeks after the 2^nd^ immunization as described [15]. **Fig.4A** shows that A20-silenced BMMфs fully protect the immunized mice from EG-7 challenge. We further tested the A20-silenced BMMф-triggered immune protection by challenging the immunized mice with a more aggressive, OVA-expressed melanoma cell line, M05. **Fig.4B** shows that A20-silenced BMMфs were still superior to control Mф in protecting the immunized mice from the M05 challenge.

Recent studies indicated that tumor-reactive CD4^+^ T cells have a potential to up-regulate expression of MHC class-II on melanoma B16 cells, and thereby reject the cells by an MHC-II restricted mechanism in a mouse model [1], [2]. To demonstrate contribution of CD4^+^ T cells to A20-silenced BMMф-triggered immune protection, OT-II-pulsed, A20-silenced BMMфs were used to immunize CD4^−/−^mice and the wildtype littermates followed by a challenge of melanoma M05 cells two weeks after the 2^nd^ immunization. **Fig.4C** shows that, in contrast to wild-type mice, which were protected from tumor occurrence with 80% efficiency, CD4^−/−^ mice only achieved 20% of protection after A20-silenced BMMф immunization.

To directly confirm cytotoxic CD4^+^ T cell-mediated immune protection, naïve C57BL/6 mice were inoculated with 6×10^5^ OVA-expressing B6SJ003 followed by adoptive transfer of 5×10^6^ in vitro primed CD4^+^ OT-II cells with OT-II-pulsed, A20-silenced BMMф or control BMMф. T cell adoptive transfer was repeated once at a one-week interval. **Fig.4D** shows that OT-II cells primed by A20-silenced BMMф are superior to those primed by control BMMф in inhibiting onset and growth of the engrafted OVA-expressed B6SJ003 tumor. However, treatment of A20-silenced BMMф/OT-II coculture with 100 nM of CMA for 1 hr prior to OT-II adoptive transfer ablates the superior ability of the OT-II cells in rejection of the engrafted tumor. Taken together, the results support that A20-silenced BMMфs not only elicit CD8^+^ T cells and NK cell to combat tumor, also effectively trigger cytotoxic CD4^+^ T cell response for anti-tumor immune protection.

### A20 Restricts Mф to Trigger Cytotoxic CD4^+^ T Cell Response by Limiting IFN-γ Production

**Figure 5:**
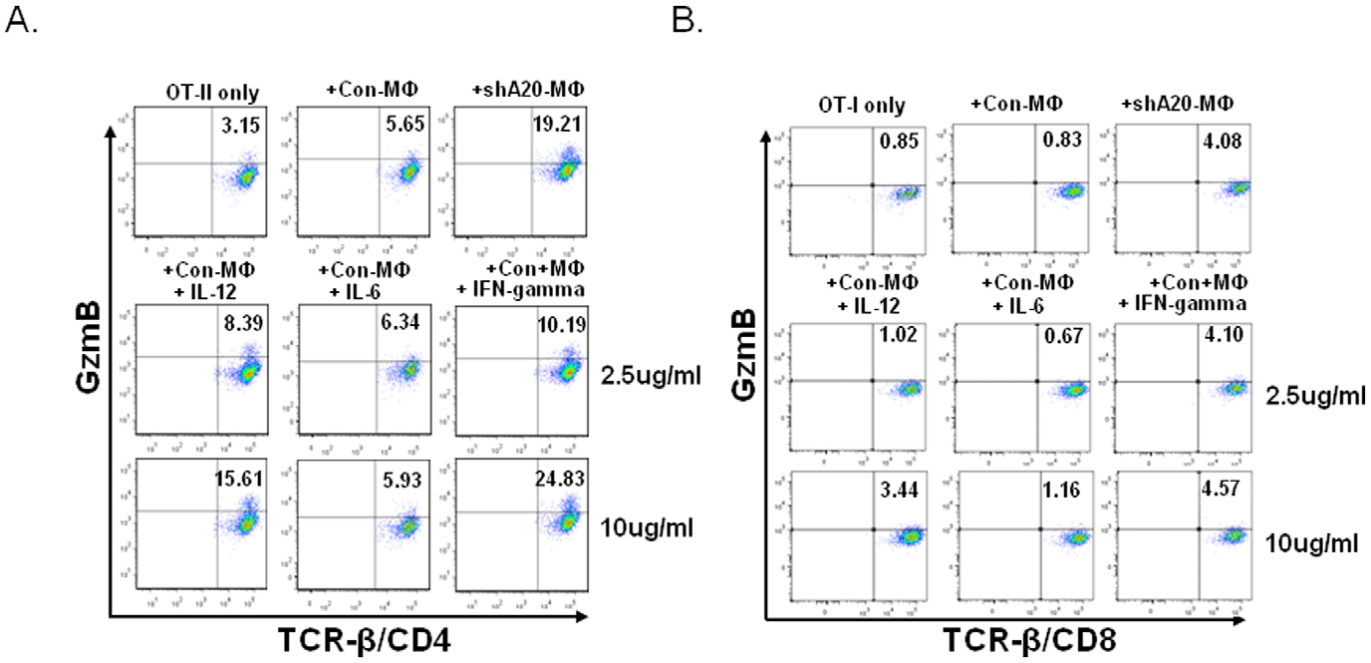
IFN-γ enhances MФ to prime cytotoxic T cells response *in vitro.* BMMфs were transduced with Ad-con and cocultured with CD4^+^ OT-II (**A**) or CD8^+^ OT-I (**B**) T cells in the presence of the different doses of IL-6, IL-12 or IFN-γ (2.5 ug/ml or 10 ug/ml ) for 3–5 days. A20-silenced MФ priming OT-II or OT-I T cells was used as positive control. Expression of granzyme B in T cells was assessed by ICS assay. The data is a representative of three independent experiments. p<0.01, OT-II/con-Mф+IFN-γ(10 ug/ml) vs. OT-II/con-Mф or OT-I/con-Mф+IFN-γ (10 ug/ml) vs. OT-I/con-Mф.

As described above, A20-silenced BMMфs not only express enhanced proinflammatory cytokines, also prime the cocultured T cells to produce higher levels of proinflammatory cytokines. To determine whether the enhanced cytokine expression relates to the distinct activity of Mф in triggering a cytotoxic CD4^+^ T cell response, the control, but not A20-silenced, BMMфs were cocultured with CD8^+^ OT-I or CD4^+^ OT-II T cells in the presence of varying doses of IFN-γ, IL-12, or IL-6. As shown in **Fig.5A**, while the addition of IL-6 did not promote BMMф to trigger granzyme B expression in the cocultured CD4^+^ OT-II cells and the addition of IL-12 promoted BMMф to trigger granzyme B expression in the cocultured CD4^+^ T cells at a medium level, addition of IFN-γ drastically enhanced BMMф to trigger granzyme B expression in the cocultured CD4^+^ T cells. Addition of IFN-γ also enhanced the ability of BMMф to trigger perforin^+^-CD4^+^ T cell response (data not shown), but the result is not so convincing likely due to the antibody’s limitation in recognizing perforin in cocultured T cells. Furthermore, addition of IFN-γ was found to endow BMMф with a comparable ability to A20-silenced BMMф in eliciting expression of granzyme B in CD8^+^ T cells, but the overall granzme B level in the cocultured CD8^+^ T cells is much lower than those in the cocultured CD4^+^ T cells (**Fig.5B** **&** **Fig. 2A** **)**. These results suggest that enhanced production of IFN-γ by A20-silenced BMMфs may contribute to priming of the cytotoxic T cells, especially to priming of cytotoxic CD4^+^ T cells.

**Figure 6:**
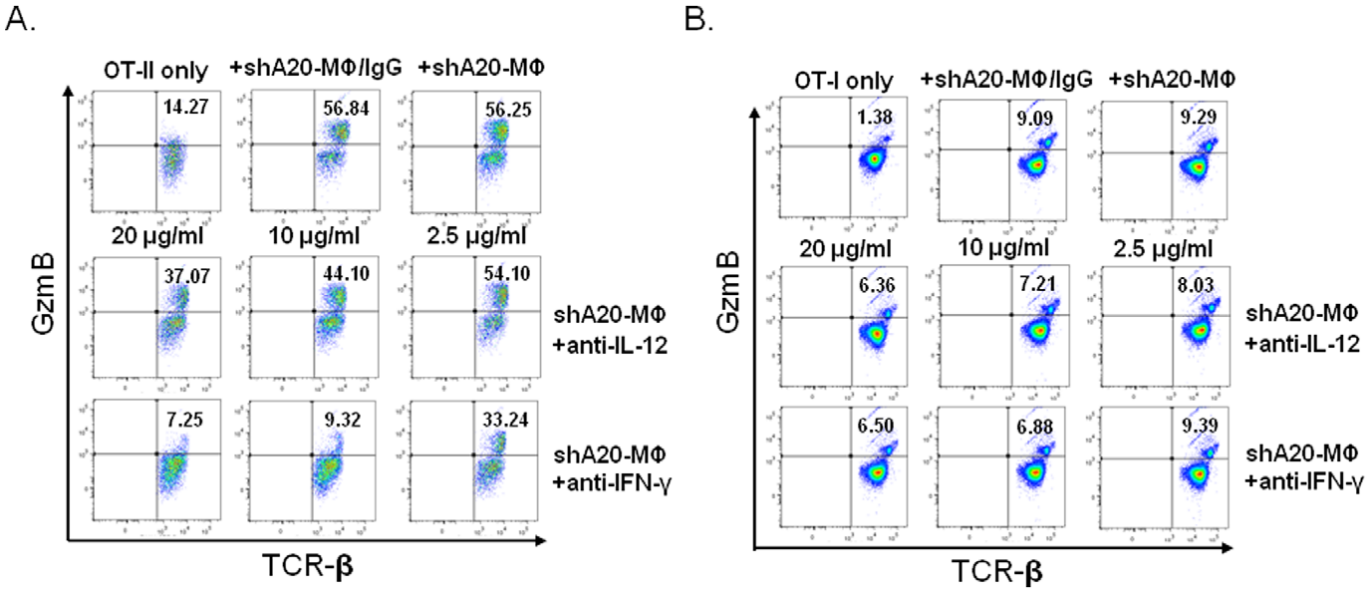
Neutralization of IFN-γ reduces A20-silenced MФ to prime cytotoxic T cell response *in vitro.* BMMфs were transduced with Ad-shA20 and cocultured with CD4^+^ OT-II (**A**) or CD8^+^ OT-I (**B**) T cells in the presence of the different doses of anti-IL-6, anti-IL-12 or anti-IFN-γ (2.5 ug/ml, 10 ug/ml, or 20 ug/ml ) for 3–5 days. Expression of granzyme B in T cells was assessed by ICS assay. The data is a representative of three independent experiments. p<0.01, OT-II/AdshA20-Mф vs. OT-II/AdshA20-Mф+anti-IFN-γ(20 ug/ml).

To verify the effect of the cytokines, the coculture of A20-silenced BMMфs with T cells was added with anti-IFN-γ or anti-IL-12 to neutralize activity of these cytokines. **Fig.6A** showed that neutralization of IFN-γ, but not IL-12, dramatically reduced A20-silenced BMMф to stimulate production of granzyme in the cocultured OT-II cells. **Fig.6B** showed that neutralization of either cytokine IL-12 or IFN-γ reduced A20-silenced BMMф to produce granzyme-expressing OT-I cells to a certain extent. As individually neutralizing IL-12 or IFN-γ does not reduce expression of the cytotoxic molecule to the level in cocultured OT-I with con-BMMфs (data not shown) or OT-I culture alone (**Fig.6B** **)**, a synergistic effect of these cytokines may be required for BMMф to optimally stimulate a cytotoxic CD8^+^ T cell response, at least on the cellular level. The results suggest that A20-silenced BMMфs provoke cytotoxic CD8^+^/CD4^+^ T cells likely through different mechanisms. A20-silenced BMMфs have a superior ability to trigger a cytotoxic CD4^+^ T cell response largely by enhancing the production of both autocrine and paracrine IFN-γ.

**Figure 7:**
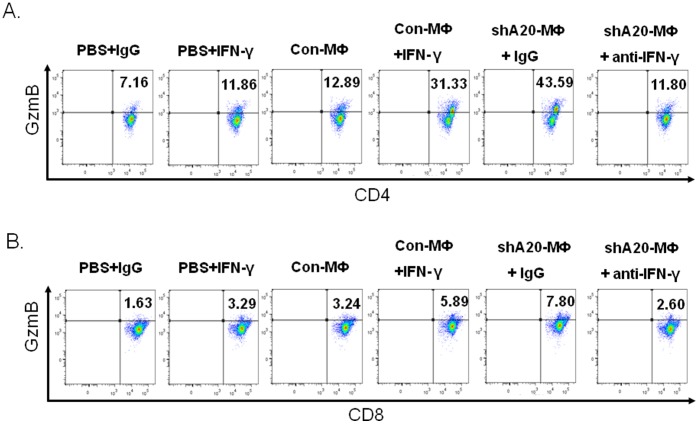
IFN-γ impacts MФ to trigger cytotoxic T cell responses in immunized mice. C57BL/6 mice (2–3 mice per group) were immunized twice with 1, PBS plus IgG; 2, PBS plus IFN-γ; 3, Ad-con-Mф; 4, Ad-con-Mф plus IFN-γ; 5, Ad-shA20-Mф plus IgG; or 6, Ad-shA20-Mф plus anti-IFN-γ. Two weeks after the 2^nd^ immunization, inguinal lymph nodes were harvested to analyze expression of granzyme B in CD4^+^ T cells (**A**) (p<0.05, shA20-Mф+ anti-IFN-γ immunization vs. shA20-Mф+IgG immunization; p<0.01, con-Mф+ IFN-γ immunization vs. con-Mф immunization) or CD8^+^ T cells (**B**) (p<0.01, shA20-Mф+ anti-IFN-γ immunization vs. shA20-Mф+IgG immunization; p<0.05, con-Mф+ IFN-γ immunization vs. con-Mф immunization) by ICS assay.

To confirm the observed in vitro effect of IFN-γ in immunized mice, groups of C57BL/6 mice were immunized twice as the indicated in **Fig.7**. All the BMMфs were pulsed with OT-I/OT-II prior to immunization. Antibody (250 ug/mouse) was administrated (i.p) one day before BMMф immunization, or IFN-γ (1 ug/mouse) administered on the same day as the BMMф immunization and two days later. ICS analysis of the inguinal LNs showed that immunization of control BMMфs with the IFN-γ co-administration dramatically activated granzyme B expression in CD4^+^ T cells, whereas, immunization of A20-silenced BMMф with the anti-IFN-γ co-administration drastically reduced granzyme B expression in these CD4^+^ T cells (**Fig. 7A**). In parallel, co-administration of IFN-γ was found to enhance control BMMф to stimulate CD8^+^ T cells, while co-injection of anti-IFN-γ attenuated A20-silenced BMMф to stimulate CD8^+^ T cell response (**Fig.7B**). Injection of IFN-γ alone did not achieve significantly cytotoxic T cell responses (**Fig.7A&B**). A similar but not identical response pattern was obtained from analysis of splenic CD4^+^/CD8^+^ T cells (**Fig.S8**). These results highlight that IFN-γ is critical for Mф to activate a cytotoxic CD4^+^ T cell response and that A20 controls Mф to activate cytotoxic T cells by limiting IFN-γ production.

### A20-silenced Mф Elicits a Cytotoxic CD4^+^ T Cell Response by Activation of IFN-γ Signaling as Well as by an MHC-class-II-restricted Mechanism

**Figure 8:**
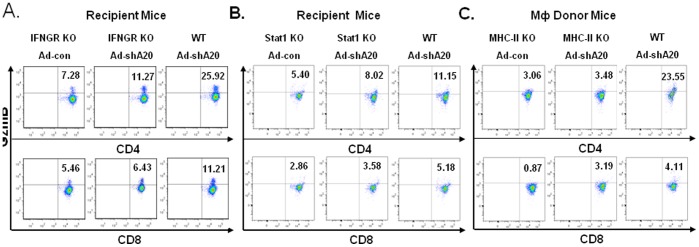
A20-silenced Mф elicits a cytotoxic CD4^+^ T cell response via activation of IFN-γ signaling and by an MHC-class-II-restricted mechanism. A. Adenoviral-transduced BMMфs were used to immunize IFNGR^−/−^ mice or the wildtype littermates (2–3 mice/group) twice. The inguinal LNs were harvested for analyzing expression of granzyme B in CD4^+^ or CD8^+^ T cells by ICS. p<0.01 Ad-shA20-IFNGR KO mice vs. Ad-ShA20 WT mice. B. Adenoviral-transduced BMMфs were used to immunize Stat1^−/−^ mice or the wild-type littermates twice (2–3 mice/group). The LNs were harvested for analyzing expression of granzyme B in CD4^+^ (p<0.05, Ad-shA20-Stat1 KO mice vs. Ad-shA20-WT mice) or CD8^+^ T cells by ICS. C. BMMфs were prepared from MHCII^−/−^ mice or the wild-type littermates. The adenoviral-transduced BMMфs were used to immunize wild-type mice (2–3 mice/group) twice. The LNs were harvested for analyzing expression of granzyme B in CD4^+^ (p<0.01, Ad-shA20-MHC-II KO Mф immunization vs. Ad-shA20-WT Mф immunization) or CD8^+^ T cells by ICS. Experiments were repeated with similar results.

IFN-γ exerts its effects on cells by interacting with a specific receptor composed of two subunits, IFNGR1 and IFNGR2, and thereby phosphorylating Jak/Stat1 signaling molecules [16]. To demonstrate A20-silenced BMMфs provoking potent cytotoxic T cell response through activation of IFN-γ signaling, A20-silenced BMMфs and control pulsed with OT-I/OT-II were used to immunize IFNR1^−/−^ mice and their wildtype littermates. ICS analysis of the inguinal LNs showed that A20-silenced BMMфs had an equivalent or higher efficacy than the control BMMфs to induce CD4^+^/CD8^+^ cytotoxic T cell responses in IFNGR1^−/−^ mice, but had a significantly lower efficacy compared with what they did in wildtype mice (**Fig. 8A**). The result implies that IFN-γ receptor is required for A20-silenced BMMф to elicit cytotoxic T cell responses, but other signaling pathways also contribute some to the function of A20-silenced BMMфs. Furthermore, A20-silenced or control BMMфs were used to immunize Stat1^−/−^ mice in parallel with their wildtype littermates. As Stat1^−/−^ mice are under the 129S background, OVA protein instead of the peptides was used to pulse the BMMф for immunization. Again, ICS showed that A20-silenced BMMф had an equivalent or higher efficacy than the control BMMф to induce CD4^+^/CD8^+^ cytotoxic T cell responses in Stat1^−/−^ mice, but the efficacy is significantly lower than what they did in wildtype mice (**Fig. 8B**), which supports that IFN-γ-triggered Stat1 signaling is required but not the only for A20-silenced BMMф to elicit cytotoxic T cell responses. Indeed, Zimmermann et al reported that IFN-γ directly activates Stat2 signaling for the antiviral potency [17]. We also analyzed splenocytes from the immunized IFNR^−/−^ mice and Stat1^−/−^ mice and obtained similar but not identical results (**Fig. S9A&B**).

Ultimately, we tested whether A20-silenced BMMф uses a MHC class-II-restricted mechanism to induce cytotoxic T cell response. BMMфs were prepared from MHCII^−/−^ mice or wildtype littermates. The OT-I/OT-II-pulsed, adenoviral-transduced BMMфs were used to immunize wildtype C57BL/6 mice as described. ICS analysis of inguinal LNs shows that A20-silenced MHCII^−/−^ Mф, equivalent to the control MHCII^−/−^ Mф, displayed a significantly lower efficacy than their wild-type counterpart in the activation of cytotoxic CD4^+^ T cells. However, A20-silenced MHCII^−/−^ Mфs barely lost their ability in activation of cytotoxic CD8^+^ T cells when compared with A20-silenced wild-type BMMфs (**Fig. 8C**). A similar but not identical result was obtained from ICS analysis of the immunized splenocytes (**Fig. S9C**). These results support that A20-silenced BMMфs activate a cytotoxic CD4^+^ T cell response in an MHC class-II restricted manner. A20 controls Mфs to activate cytotoxic T cell responses largely by limiting IFN-γ signaling.

## Discussion

Cytotoxic CD4^+^ T cells were detected in both mouse and human over 20 years ago. The early evidence claimed that distinct from cytotoxic CD8^+^ T cells, CD4^+^ T cells use the FAS/FAS ligand system for the cytolytic activity [18], [19]. Recent studies strongly supported that granule exocytosis of perforin/granzymes represents the main pathway of cytotoxicity in both CD4^+^ and CD8^+^ T cells [20], [21], [22], [23], [24], [25]. In line with these studies, our study suggested that granzyme B as well as possible perforin can be induced in CD4^+^ T cells by A20-silenced Mфs and the resultant CD4^+^ T cells rejected engrafted tumors in a perforin/granzyme-dependent manner. Although freshly isolated CD4^+^ T cells from A20-silenced Mф immunized mice display some nonspecific cytotoxicity, the isolated CD4^+^ T cells after in vitro re-stimulation use MHC class-II restricted mechanism to kill tumor cells. CD4^+^ T cell killing of infected or malignant cells in MHC-class II-restricted manner has been reported in several studies [23]. Quezada et al. and Xie et al. recently further claimed that tumor-reactive CD4^+^ T cells secrete a copious amount of IFN-γ to upregulate expression of MHC-class-II molecules on tumor cells and make them the target of cytotoxic CD4^+^ T cells after transfer into lymphopenic hosts [1], [2]. Thus, our reported, A20-silenced Mф induced, CD4^+^ T cells exhibit common functional features to those in vivo or ex vivo differentiated cytotoxic CD4^+^ T cells. It is worth mentioning here that throughout the whole study, we persistently detected a higher level of perforin in either stimulated or immunized T cells by A20-silenced Mфs and the expressing pattern of perforin in these T cells resembled the expression of granzyme B, but the results may not be convincing due to the antibodies’ limitation.

Cytotoxic CD4^+^ T cell differentiation occurs under different physiological or pathological conditions. Recent studies further investigated cytotoxic CD4^+^ T cells by adoptive cellular transfer (ACT) of antigen-specific CD4^+^ T cells or creation of antigen-specific TCR-transgenic mice. Brown et al. explored that virus-specific TCR transgenic CD4^+^ cells acquired perforin-mediated cytolytic activity after adoptive transfer into influenza-infected mice, and that the perforin-dependent cytolysis represents one of the important mechanisms to protect mice from lethal influenza infection [26]. Xie et al. and Quzezada et al. reported that naïve tumor-specific CD4^+^ T cells develop cytotoxic activity and eradicated established melanoma after transfer into lymphopenic hosts [1], [2]. Corthay et al. unveiled that primary antitumor immune response can be triggered by transgenic ID-specific CD4^+^ T cells in immune deficient SCID mice [27]. All these studies revealed a dominant type-I immune response environment associated with the cytotoxic CD4^+^ T cell differentiation. For example, EBV-specific CD4^+^ T cells represent one of the earliest defined cytolytic CD4^+^ T lymphocytes. Paludan et al. reported that EBV infection triggers CD4^+^ T cell to primarily differentiate into IFN-γ-producing Th1-type [28]. Xie et al and Quzezada et al adoptively transferred tumor antigen-specific CD4^+^ T cells into lymphopenic mice. Their studies also claimed that Th1 polarization is a default pathway in lymphopenic host [1], [2]. Corthay et al found that transgenic ID-specific CD4^+^ T cells infiltrate into tumors and produce Th1 cytokines in mice with an immune deficient background [27]. Recently, Muranski et al. discovered that Th17-polarized tumor-reactive CD4^+^ T cells are capable of rejecting established melanomas [29]. Their subsequent study informed that Th17 cells are metastable and able to gradually acquire a Th1-like phenotype secreting less IL-17A and more IFN-γ [30]. Our reported A20-silenced Mфs produce high levels of proinflammatory cytokines and preferentially prime IFN-γ/TNF-α-producing T cells, which further supports type-I immune environment promotes cytotoxic CD4^+^ T cell development.

Our study further defined that IFN-γ is crucial for A20-silenced Mф to induce cytotoxic CD4^+^ T cell differentiation. IFN-γ impact on cytotoxic CD4^+^ T cell responses has been implicated in many published studies. Mumberg et al. reported that anti-IFN-γ treatment abolishes the CD4^+^ T cell-mediated rejection of the tumor cells in SCID mice [31]. Corthay explored that CD4^+^ T cells mediate tumor rejection by producing IFN-γ to activate Mф-associated antitumor activity [27]. Perez-diez et al. revealed that CD4^+^ T cells obtain the maximal antitumor effect by partnering with NK cells, an innate source of IFN-γ [32]. Furthermore, both Xie et al. and Quezada et al. defined that IFN-γ facilitates cytotoxic CD4^+^ T cells to reject malenoma by up-regulation of MHC class-II expression on tumor cells [1], [2]. In our present study, IFN-γ is found to directly promote expression of cytotoxic molecules in CD4^+^ T cells, which is consistent with an early report that activation of IFN signaling was required for expression of perforin and granzyme in CD8^+^ T cells and NK cells in melanoma patients [33]. Thus, IFN-γ exhibits comprehensive functions associated with cytotoxic CD4^+^ T cell response, while our present result suggested a novel mechanism for IFN-γ functioning CD4^+^ T cell-mediated cytotoxicity. Our study further indicated that A20-silenced Mф-induced cytotoxic CD4^+^ T cell differentiation is MHC class-II restricted, which coincides with published studies that tumor-reactive CD4^+^ T cells develop cytotoxic activity in an MHC class-II-dependent manner [34] and priming of tumor-reactive CD4^+^ T cells requires MHC class-II expression on recipient or host cells, not on tumor cells [1], [2], [27]. Most intriguingly, Corthay et al identified that tumor infiltrated macrophages are an important component to re-activate tumor-specific CD4^+^ T cells by presenting tumor-derived peptides on their MHC-II molecules [27]. Our study further suggested that the re-activation step also triggers CD4^+^ T to express and exocytose cytotoxic molecules for directly killing MHC-II-restricted tumor cells and MHC-II-non-restricted tumor cells in the close proximity.

Ex vivo generated, tumor-reactive, autologous CD4^+^ T cell clones have successfully been used to treat melanoma patients [35]. Our study may provide a platform for in vitro generating antigen-specific cytotoxic CD4^+^ T cells for adoptive tumor immunotherapy.

## Methods

### Mice

C57BL/6, H-2K^b^/OT-I–TCR (OT-I) transgenic mice, H-2K^b^/OT-II–TCR (OT-II) transgenic mice, CD4 knockout (CD4^−/−^) mice, IFNGR1 knockout (INFGR^−/−^) mice, MHC class-II knockout mice (MHCII^−/−^), and Stat1 knockout (Stat1^−/−^) mice were purchased from Jackson Laboratories or Taconic Farms. All the mice were maintained in a mouse facility at USC according to institutional guidelines. This study was approved by the Institutional Animal Care and Use Committee of USC.

### Peptides, Proteins and Cell Lines

H2-K^b^–restricted OT-I and OT-II peptides were synthesized by Genemed Synthesis. OVA protein was purchased from Sigma-Aldrich. The B6SJ003 Burkitt lymphoma cell line (H2-K^b^, MHC-II-expressed) was kindly provided by Herbert C. Morse III at the NIAID/NIH [36]. OVA-expressing B6S1003 was generated by stable transfection of OVA gene. B16-OVA melanoma cell line M05 (H2-K^b^) was kindly provided by R. Dutton at the Trudeau Institute [37]. Lymphoma cell EG-7 (H2-K^b^) which engineeringly expresses OVA was purchased from ATCC.

### Mф Immunization and Tumor Models

Mouse BMMфs were generated by culturing BM cells in the presence of macrophage colony-stimulating factor (M-CSF). The differentiated BMMфs were incubated with Ad-shA20 or Ad-con at a multiplicity of infection (MOI) of 500, which allows ∼60% of Mфs to be transduced as demonstrated by Ad-GFP transduction of Mф in parallel (data not shown). The transduced Mфs were pulsed with H2-K^b^-restricted OT-I or OT-II peptide, followed by ex vivo maturation with LPS (100 ng/ml). The Mфs (0.5–1×10^6^) were then i.p. injected into C57BL/6 mice twice at a one-week interval. For tumor challenge, two weeks after the 2^nd^ immunization, the mice received s.c. injection of 5×10^5^ EG-7 or M05. Tumor onset and growth were monitored weekly.

### In vitro T Cell Priming

T cells were purified from OT-I or OT-II transgenic mice using the MACS CD8^+^ or CD4^+^ T cell isolation kits (Miltenyi Biotec). 5×10^4^ purified T cells and 5×10^3^ adenoviral-transduced, OT-I or OT-II peptide-pulsed BMMф were cocultured in RPMI 1640 medium supplemented with 10 U/ml of IL-2. In some experiments, anti-IFN-γ or anti-IL-12 was added into the co-cultures at the final concentration of 2.5 ug/ml, 10 ug/ml, or 20 ug/ml, or IFN-γ, IL-12 or IL-6 was added at the final concentration of 2.5 ug/ml or 10 ug/ml. After 3–5 days of coculture, T cells were harvested to analyze the indicated cytokines by ICS assay.

### Adoptively Transfer Assay

The isolated OT-II cells were cocultured with adenoviral-transduced, OT-II peptide-pulsed BMMфs for 3–5 days at Mф:T ratio of 1∶10. The cocultured OT-II cells (5×10^6^) were harvested and transplanted into naïve RAG^−/−^C57BL/6 mice by retro-orbital injection followed by tumor challenge. The transplantation of OT-II T cells was repeated one week later.

### Flow Cytometric Analysis

For ICS assay, lymphocytes were harvested from draining lymph nodes or spleens of immunized mice and cultured with 20 ug/ml of OT-I or OT-II peptide for 6–10 hours at 37°C in the presence of GolgiPlug (BD Biosciences/Pharmingen). After surface staining with anti-CD8 or anti-CD4, cells were permeabilized and stained for intracellular cytokines, as previously described [38], [39]. All the antibodies and matched isotype controls were purchased from BD PharMingen or eBioscience. Stained cells were analyzed on a FACSaria (Becton Dickinson) flow cytometer and FloJo software.

### CTL and NK Assays

Different numbers of effector cells (5×10^5^, 2.5×10^5^ or 1.25×10^5^) were cocultured with a certain number (5000 cells) of Yac-1 (for NK assay), EG-7 (for CD8^+^ T cell assay), or OVA-expressed B6SJ1003 (for CD4^+^ T cell assay) for 5 hrs. EL-4 tumor cell line was used as a non-specific control. Some of the cocultures were added with 3 nM CMA or 1 mM EGTA to inhibit activity of perforin and granzyme. The supernatants were harvested and analyzed by LDH release assay (Roche Diagnostics).

### Statistical Analysis

We used the Student’s t-test. A 95% confidence limit was used to assess results for statistical significance, defined as *P*<0.05. Results are typically presented as means ± standard error.

## Supporting Information

Figure S1 **Ad-shA20 reduces expression of A20 mRNA in transduced BMMф.** BMMфs were transduced with Ad-shA20, Ad-con, or PBS. 24 hr later, the Mфs were stimulated with 100 ng/ml LPS or none for overnight. **A**, relative expression of A20 mRNA in the transduced BMMфs was evaluated by qRT-PCR. * p<0.05, Ad-shA20- Mф vs. Ad-con-Mф. **B**, A20 protein expression in the transduced BMMфs was evaluated by ICS. The anti-A20 was purchased from Santa Cruz. Experiments were repeated twice with similar results.
(TIF)

Figure S2 **Ad-shA20 barely enhances apoptosis of the transduced BMMфs.** BMMфs were transduced with Ad-shA20 or Ad-con. 24 hr later, the Mфs were stimulated with PBS, anti-CD40 (10 ug/ml), or LPS (100 ng/ml) for overnight. The treated BMMфs were analyzed with Annexin V-APC Apoptosis Detection Kit (BD Bioscience). Experiments were repeated with similar results.
(TIF)

Figure S3 **A20-silenced Mф promotes proinflammatory status of the cocultured OT-I T cells.** The adenoviral-transduced Mфs were cocultured with freshly isolated OT-I T cells in the presence of OT-I peptide at the ratio of 1 to 10. 3–5 days later, the OT-I T cells were harvested and analyzed for expression of surface markers CD25, CD69, CD44, and CD62L by cell surface staining and for production of proinflammatory cytokines IFN-γ and TNF-α by ICS. Experiments were repeated with similar results.
(TIF)

Figure S4 **A20-silenced Mф promotes proinflammatory status of the cocultured OT-II T cells.** The adenoviral-transduced Mфs were cocultured with freshly isolated OT-II T cells in the presence of OT-II peptide at the ratio of 1 to 10. 3–5 days later, the OT-II T cells were harvested and analyzed for expression of surface markers CD25 and CD69 by cell surface staining, and for production of inflammatory cytokines IFN-γ, TNF-α and IL-4, as well as transcription factor FoxP3 by ICS. Experiments were repeated with similar results.
(TIF)

Figure S5 **A20-silenced Mф enhances expression of perforin in CD4^+^ T cells, CD8^+^T cells or NK cells. A,** adenoviral-transduced Mфs were cocultured with freshly isolated OT-I (**uppe**r) or OT-II cells (**lower**) at a raito of 1∶10. 3–5 days later, the cocultured T cells were harvested for analyzing expression of proferin by ICS. The data is shown as a representative of 3 independent experiments. **B,** C57BL/6 mice (5–6 mice/group) were immunized (*i.p*) twice with different adenoviral-transduced Mфs or PBS. Lymphocytes were isolated from the inguinal LNs to analyze expression of proferin in NK cells, CD8**^+^** or CD4**^+^** T cells by ICS. The data is shown as a representation of three independent experiments.
(TIF)

Figure S6 **pshuttle-shA20-transfected Mфs prime cytotoxic OT-II T cell response in vitro.** BMMфs were neuclofected with pshuttle-shGFP or pshuttle-shA20. 24 hrs later, the transfected BMMфs were cocultured with freshly isolated OT-II T cells in the presence of OT-II peptide for 3–5 days. OT-II T cells were harvested for analyzing expression of granzyme B and perforin by ICS. Experiment was repeated once with similar results.
(TIF)

Figure S7 **Z-AAD-CMK inhibited CTL activity mediated by A20-silenced Mф-immunzed CD4^+^ T cells.** OT-II (not OT-I)-pulsed, differently transduced BMMфs were used to immunize C57BL/6 mice and splenocytes were harvested and restimulated with OT-II peptide for 5–6 days. Various ratios of the splenocytes and target cells (OVA-expressing B6SJ003) were cocultured with or without 75 uM of Z-AAD-CMK for 6 hrs. Cytotoxic activities were analyzed by LDH release assay as described in Material and Methods. Experiments were repeated once. *p<0.05, Ad-shA20-Mф immunization vs. Ad-shA20-Mф immunization plus the Z-AAD-CMK treatment.
(TIF)

Figure S8 **IFN-γ impacts MФ to trigger cytotoxic T cell responses in immunized mice.** C57BL/6 mice were immunized twice with 1, PBS plus IgG; 2, PBS plus IFN-γ; 3, Ad-con-Mф; 4, Ad-con-Mф plus IFN-γ; 5, Ad-shA20-Mф plus IgG; or 6, Ad-shA20-Mф plus anti-IFN-γ. Antibody (250 ug/mouse) was i.p administrated one day before Mф immunization, and IFN-γ (1 ug/mouse) was given on the same day as the Mф immunization and two days later. Two weeks after the 2^nd^ immunization, splenocytes were harvested for intracelluar granzyme staining of CD4 T cells (**A**) or CD8 T cells (**B**).
(TIF)

Figure S9 **A20-silenced Mф elicits a cytotoxic CD4^+^ T cell response via activation of IFN-γ signaling and by an MHC-class-II-restricted mechanism.** A. Adenoviral-transduced BMMфs were used to immunize IFNGR^−/−^ mice or the wild-type littermates twice. Splenocytes were harvested for analyzing expression of granzyme B in CD4^+^ or CD8^+^ T cells by ICS. B. Adenoviral-transduced BMMфs were used to immunize Stat1^−/−^ mice or the wild-type littermates twice. Splenocytes were harvested for analyzing expression of granzyme B in CD4^+^ or CD8^+^ T cells by ICS. C. BMMфs were prepared from MHCII^−/−^ mice or wild-type littermates. The adenoviral-transduced BMMфs were used to immunize wild-type mice twice. Splenocytes were harvested for analyzing expression of granzyme B in CD4^+^ or CD8^+^ T cells by ICS. Experiments were repeated with similar results.
(TIF)
