# Supplementary figures and images for: A20 Controls Macrophage to Elicit Potent Cytotoxic CD4+ T Cell Response
Source: PLoS One. 2012 Nov 7;7(11):e48930. doi: 10.1371/journal.pone.0048930 (PMC3492139; doi:10.1371/journal.pone.0048930)

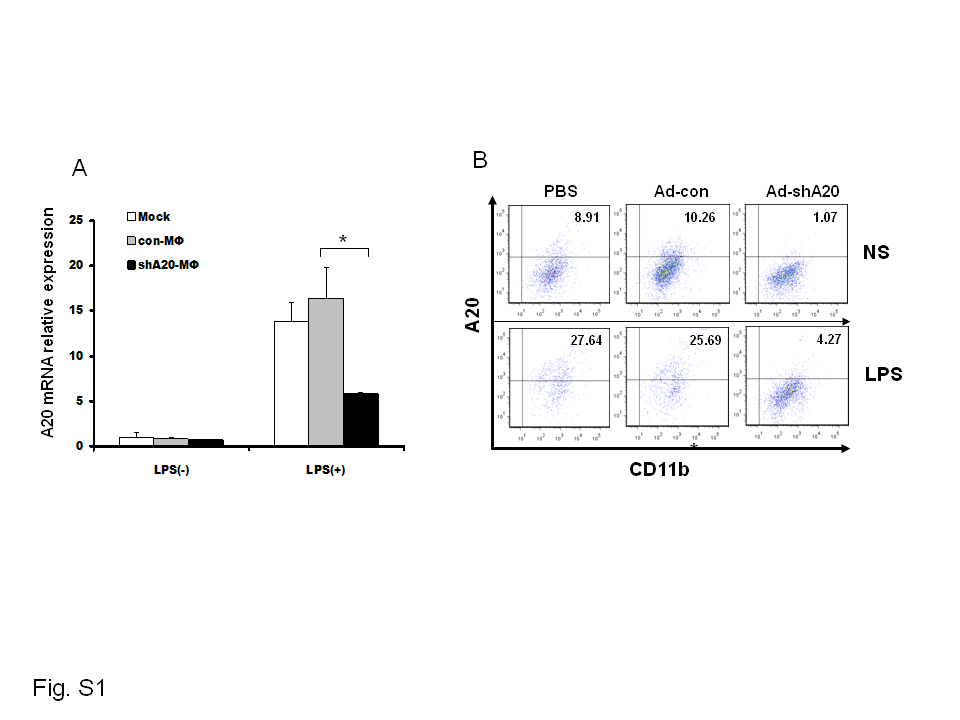

Supplement: Figure S1 — Ad-shA20 reduces expression of A20 mRNA in transduced BMMф. BMMфs were transduced with Ad-shA20, Ad-con, or PBS. 24 hr later, the Mфs were stimulated with 100 ng/ml LPS or none for overnight. A, relative expression of A20 mRNA in the transduced BMMфs was evaluated by qRT-PCR. * p<0.05, Ad-shA20- Mф vs. Ad-con-Mф. B, A20 protein expression in the transduced BMMфs was evaluated by ICS. The anti-A20 was purchased from Santa Cruz. Experiments were repeated twice with similar results. (TIF) [file pone.0048930.s001.tif]

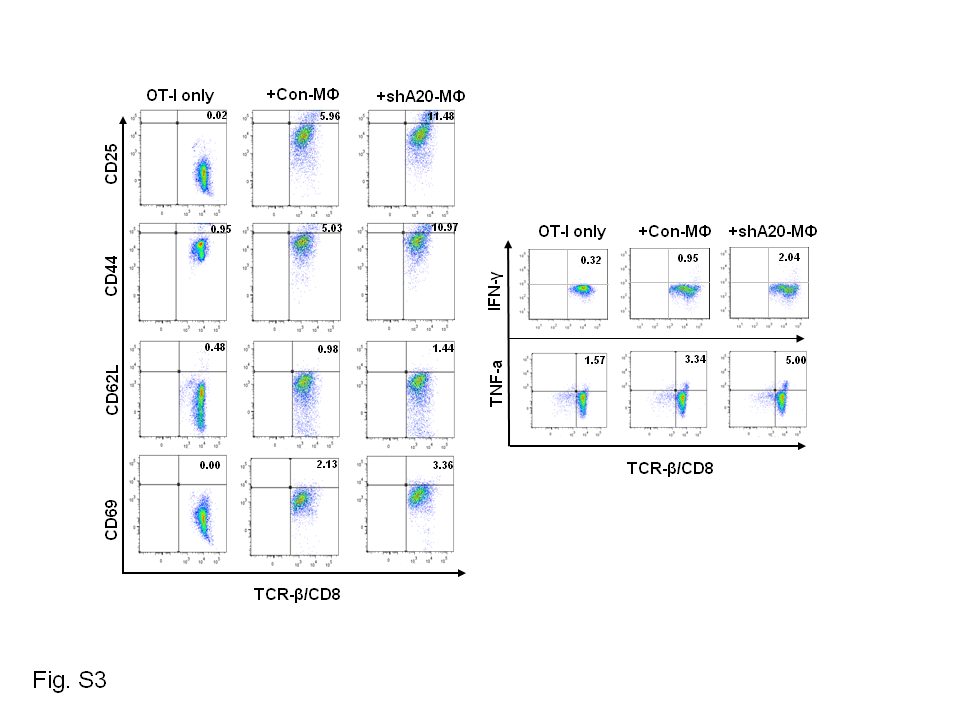

Supplement: Figure S3 — A20-silenced Mф promotes proinflammatory status of the cocultured OT-I T cells. The adenoviral-transduced Mфs were cocultured with freshly isolated OT-I T cells in the presence of OT-I peptide at the ratio of 1 to 10. 3–5 days later, the OT-I T cells were harvested and analyzed for expression of surface markers CD25, CD69, CD44, and CD62L by cell surface staining and for production of proinflammatory cytokines IFN-γ and TNF-α by ICS. Experiments were repeated with similar results. (TIF) [file pone.0048930.s003.tif]

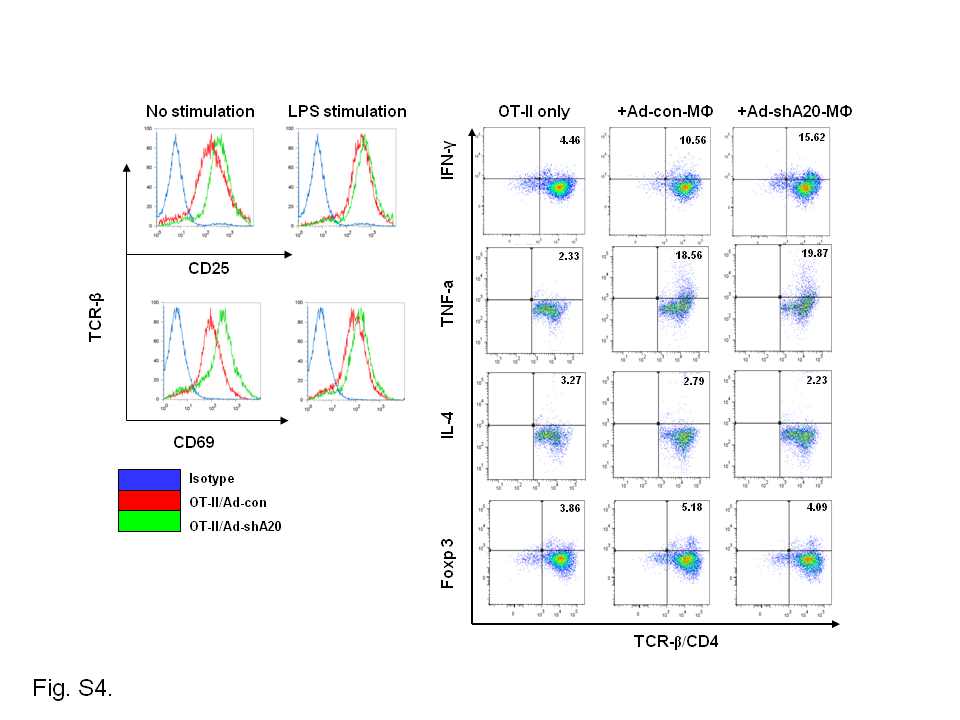

Supplement: Figure S4 — A20-silenced Mф promotes proinflammatory status of the cocultured OT-II T cells. The adenoviral-transduced Mфs were cocultured with freshly isolated OT-II T cells in the presence of OT-II peptide at the ratio of 1 to 10. 3–5 days later, the OT-II T cells were harvested and analyzed for expression of surface markers CD25 and CD69 by cell surface staining, and for production of inflammatory cytokines IFN-γ, TNF-α and IL-4, as well as transcription factor FoxP3 by ICS. Experiments were repeated with similar results. (TIF) [file pone.0048930.s004.tif]

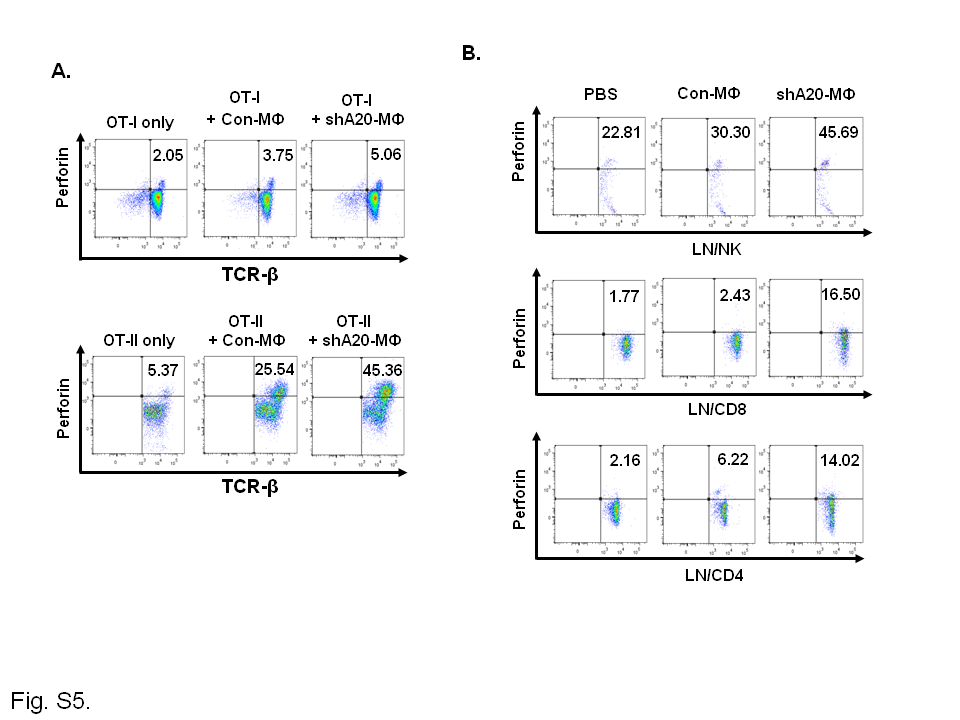

Supplement: Figure S5 — A20-silenced Mф enhances expression of perforin in CD4+ T cells, CD8+T cells or NK cells. A, adenoviral-transduced Mфs were cocultured with freshly isolated OT-I (upper) or OT-II cells (lower) at a raito of 1∶10. 3–5 days later, the cocultured T cells were harvested for analyzing expression of proferin by ICS. The data is shown as a representative of 3 independent experiments. B, C57BL/6 mice (5–6 mice/group) were immunized (i.p) twice with different adenoviral-transduced Mфs or PBS. Lymphocytes were isolated from the inguinal LNs to analyze expression of proferin in NK cells, CD8+ or CD4+ T cells by ICS. The data is shown as a representation of three independent experiments. (TIF) [file pone.0048930.s005.tif]

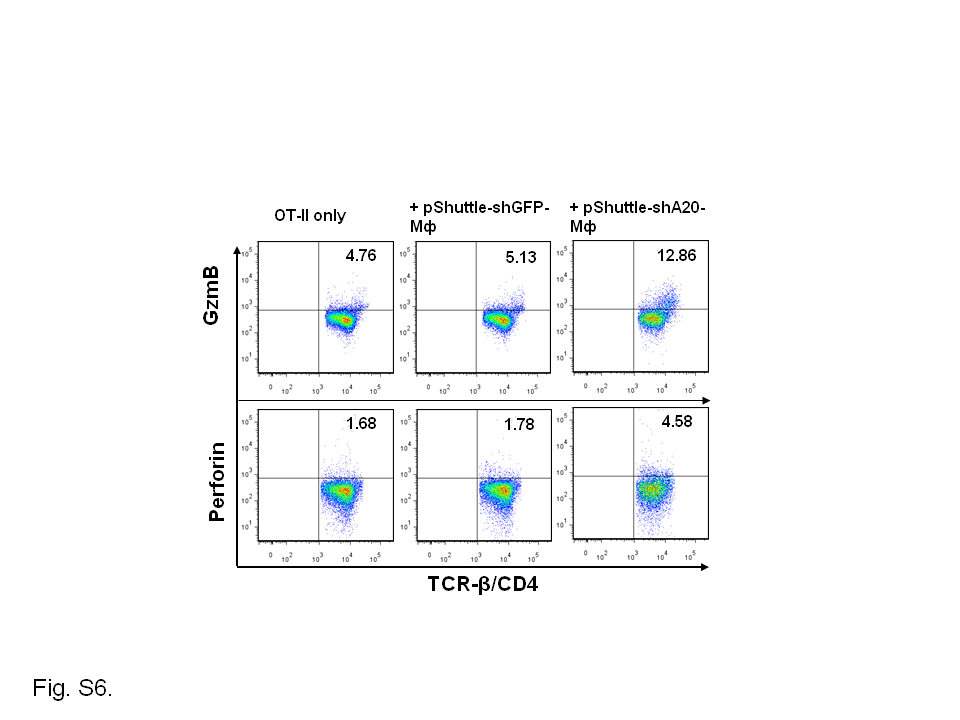

Supplement: Figure S6 — pshuttle-shA20-transfected Mфs prime cytotoxic OT-II T cell response in vitro. BMMфs were neuclofected with pshuttle-shGFP or pshuttle-shA20. 24 hrs later, the transfected BMMфs were cocultured with freshly isolated OT-II T cells in the presence of OT-II peptide for 3–5 days. OT-II T cells were harvested for analyzing expression of granzyme B and perforin by ICS. Experiment was repeated once with similar results. (TIF) [file pone.0048930.s006.tif]

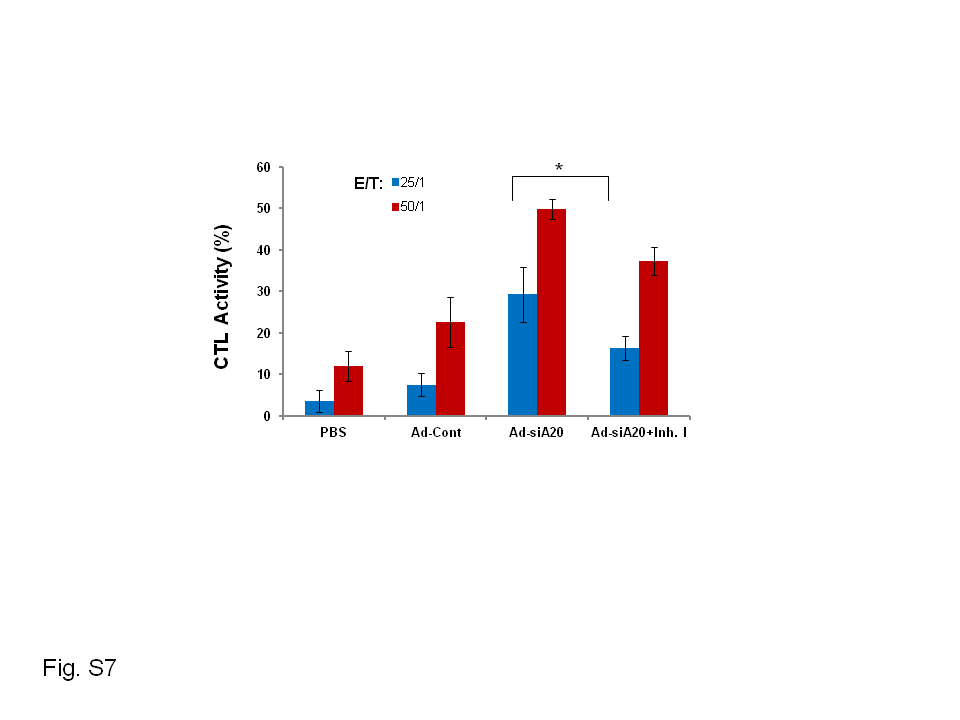

Supplement: Figure S7 — Z-AAD-CMK inhibited CTL activity mediated by A20-silenced Mф-immunzed CD4+ T cells. OT-II (not OT-I)-pulsed, differently transduced BMMфs were used to immunize C57BL/6 mice and splenocytes were harvested and restimulated with OT-II peptide for 5–6 days. Various ratios of the splenocytes and target cells (OVA-expressing B6SJ003) were cocultured with or without 75 uM of Z-AAD-CMK for 6 hrs. Cytotoxic activities were analyzed by LDH release assay as described in Material and Methods. Experiments were repeated once. *p<0.05, Ad-shA20-Mф immunization vs. Ad-shA20-Mф immunization plus the Z-AAD-CMK treatment. (TIF) [file pone.0048930.s007.tif]

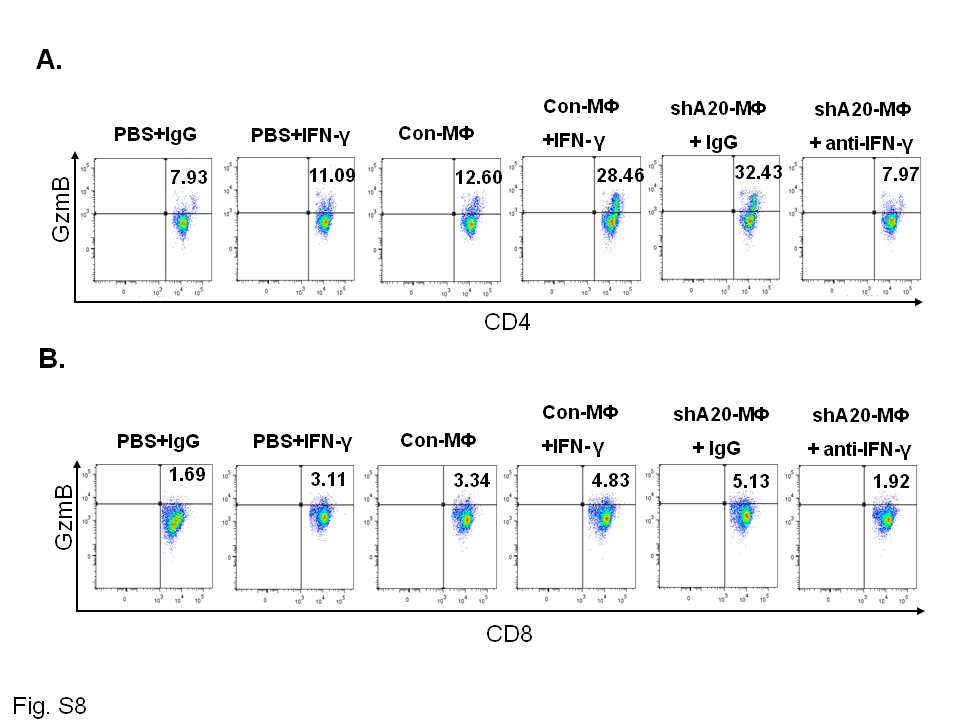

Supplement: Figure S8 — IFN-γ impacts MФ to trigger cytotoxic T cell responses in immunized mice. C57BL/6 mice were immunized twice with 1, PBS plus IgG; 2, PBS plus IFN-γ; 3, Ad-con-Mф; 4, Ad-con-Mф plus IFN-γ; 5, Ad-shA20-Mф plus IgG; or 6, Ad-shA20-Mф plus anti-IFN-γ. Antibody (250 ug/mouse) was i.p administrated one day before Mф immunization, and IFN-γ (1 ug/mouse) was given on the same day as the Mф immunization and two days later. Two weeks after the 2nd immunization, splenocytes were harvested for intracelluar granzyme staining of CD4 T cells (A) or CD8 T cells (B). (TIF) [file pone.0048930.s008.tif]

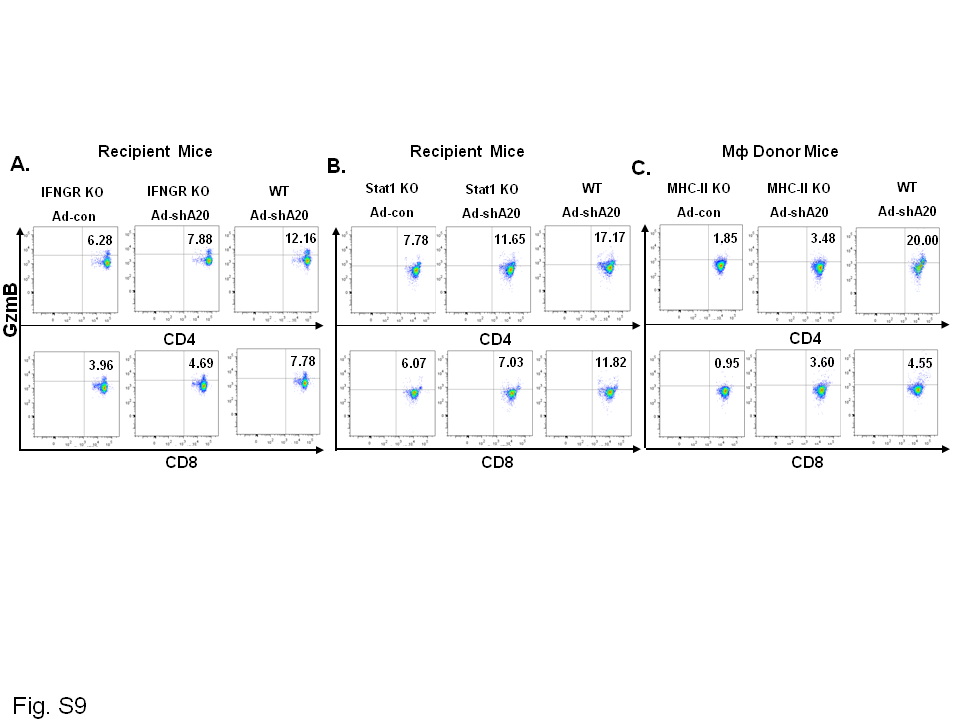

Supplement: Figure S9 — A20-silenced Mф elicits a cytotoxic CD4+ T cell response via activation of IFN-γ signaling and by an MHC-class-II-restricted mechanism. A. Adenoviral-transduced BMMфs were used to immunize IFNGR−/− mice or the wild-type littermates twice. Splenocytes were harvested for analyzing expression of granzyme B in CD4+ or CD8+ T cells by ICS. B. Adenoviral-transduced BMMфs were used to immunize Stat1−/− mice or the wild-type littermates twice. Splenocytes were harvested for analyzing expression of granzyme B in CD4+ or CD8+ T cells by ICS. C. BMMфs were prepared from MHCII−/− mice or wild-type littermates. The adenoviral-transduced BMMфs were used to immunize wild-type mice twice. Splenocytes were harvested for analyzing expression of granzyme B in CD4+ or CD8+ T cells by ICS. Experiments were repeated with similar results. (TIF) [file pone.0048930.s009.tif]
